# Mechanisms of brain overgrowth in autism spectrum disorder with macrocephaly

**DOI:** 10.3389/fnins.2025.1586550

**Published:** 2025-06-06

**Authors:** Laura Currey, Tracey Harvey, Alexandra Pelenyi, Michael Piper, Stefan Thor

**Affiliations:** School of Biomedical Sciences, University of Queensland, St Lucia, QLD, Australia

**Keywords:** ASD, megalencephaly, overgrowth, neurogenesis, autism (autism spectrum disorders)

## Abstract

Autism spectrum disorder (ASD) is a highly prevalent human disorder with extensive clinical and genetic heterogeneity. One notable ASD subgroup that often manifest with more severe symptoms comprises individuals with an enlarged head (macrocephaly), often accompanied by an enlarged brain (megalencephaly). Here, we focus on the macrocephalic ASD subgroup and discuss the biological processes that may underlie brain overgrowth in individuals with ASD, including excess neurogenesis or gliogenesis, decreased cell death, neuronal hypertrophy, and elevated myelination. We also discuss the signaling and epigenetic pathways implicated in macrocephalic ASD. By examining the biological processes and the molecular pathways involved we seek to provide insight into the mechanisms underpinning macrocephalic ASD.

## 1 Introduction

Autism spectrum disorder (ASD) is a neurodevelopmental disorder characterized by challenges in social interaction, sensory sensitivities, and repetitive behaviors. Recent meta-analysis of 71 ASD prevalence studies from 2012 to 2021 pointed to a global prevalence of around 1/100, with increasing prevalence over time (Zeidan et al., 2022). ASD also displays striking sexual differences in prevalence, with a male-to-female ratio of 4.2:1 (Zeidan et al., 2022). A continuing challenge toward diagnosing and treating ASD is its variability regarding clinical manifestations, pathology, aetiology, and sociodemographic prevalence (Zeidan et al., 2022).

In accordance with its name, ASD presents along a spectrum that encompasses a wide range of conditions with varying severity (Lord et al., 2020). ASD is prevalent in a range of other disorders, such as fragile X syndrome, Down’s syndrome, Duchenne’s muscular dystrophy, neurofibromatosis type 1 and tuberous sclerosis (Al-Beltagi, 2021). ASD is also comorbid with anxiety, attention deficit hyperactivity disorder, oppositional defiant disorder or other mood disorders (Brookman-Frazee et al., 2018), as well as sleep, gastrointestinal, metabolic, or immune disorders (Al-Beltagi, 2021). Common theories on the underlying pathology of ASD include imbalance of excitatory and inhibitory processing (Oblak et al., 2011; Rubenstein and Merzenich, 2003), connectivity deficits, such as long-range hypoconnectivity and short-range hyperconnectivity (Geschwind and Levitt, 2007; Haberl et al., 2015), and synaptic dysfunction (Guang et al., 2018).

Genetics plays a major role in ASD but is highly complex, with hundreds of genes identified as contributing to ASD (Iakoucheva et al., 2019; Vorstman et al., 2017). *De novo* mutations, including copy number variants (CNVs), account for a proportion of ASD cases (Sebat et al., 2007; Trost et al., 2022). Exome sequencing studies have identified 100’s of ASD risk genes and loci (Satterstrom et al., 2020; Sanders et al., 2015; De Rubeis et al., 2014; Iossifov et al., 2014). Additionally, common genetic variants likely contribute substantially to ASD susceptibility and account for a large proportion of ASD cases (Grove et al., 2019; Gaugler et al., 2014). Common genetic variants likely have minor impact individually, but when combined may contribute greatly to ASD, as has been observed in other neurological disorders such as schizophrenia (Ripke et al., 2014). Common variants in ASD are only recently being identified due to previous issues with small sample sizes (Grove et al., 2019), and have been aided by the increased capacity of genetic analysis. The emerging genetic landscape of ASD is being collated by the Simons Foundation Autism Research Initiative (SFARI), which currently lists 1,231 genes as implicated in ASD, sub-grouping them into “syndromic” (298 genes), “high confidence” (233 genes), “strong candidate” (708 genes) and “suggestive evidence” (156 genes).^1^ Some ASD cases have a monogenetic cause, often associated with syndromes such as Fragile X, RETT or Tuberous Sclerosis syndromes. However, no single gene accounts for more than 1% of the total number of ASD cases. Most ASD cases are genetically undefined and likely involve numerous genes.

While ASD is multifaceted and affects many tissues, many high-confidence ASD risk genes have peak expression during prenatal development, being highly expressed in the cerebral cortex, striatum, hippocampus, and cerebellum, indicating that these are critical regions and periods for the pathology of ASD. Moreover, most ASD risk genes are highly expressed in developing excitatory and inhibitory neurons (Satterstrom et al., 2020; Courchesne et al., 2019; Polioudakis et al., 2019). Because of the typical childhood stage of diagnosis, RNA-seq studies on ASD have focused on postnatal periods, revealing that gene expression is altered across most cell types in ASD cortical tissue (Parikshak et al., 2013; Voineagu et al., 2011). However, especially affected were excitatory neurons, which display decreased expression of synaptic genes, and glia, which show upregulation of proinflammatory pathways (Gandal et al., 2022; Wamsley et al., 2024; Velmeshev et al., 2019).

The high prevalence of ASD is logically mirrored by its symptomatic, pathologic, and genetic diversity, and supports the notion that ASD is likely to represent a set of related sub-disorders. Increased stratification will likely aid in uncovering the underlying pathology and genetics, allowing for more targeted diagnosis and treatment. On this note, approximately 20% of children with ASD have an enlarged head (macrocephaly) (Miles et al., 2000; Lainhart et al., 2006; Fombonne et al., 1999; Sacco et al., 2007). Moreover, ASD patients with macrocephaly exhibit more severe disability than those with normal head size, evident from lower IQ and reduced IQ increase during childhood (Amaral et al., 2017), delayed onset of language (Lainhart et al., 2006), and severity of social deficits (Hazlett et al., 2017).

In this review, we will focus on the macrocephalic ASD subgroup and discuss the biological processes and molecular pathways potentially involved.

## 2 Macrocephaly is a subgroup of ASD

Macrocephaly is typically diagnosed as head circumference above the 97th percentile, i.e., larger than 97% of children of the same age. Macrocephaly affects approximately 20% of ASD cases (Miles et al., 2000; Lainhart et al., 2006; Fombonne et al., 1999; Sacco et al., 2007; Deutsch and Joseph, 2003). By contrast, while a smaller head circumference (microcephaly) can also occur in ASD, it is less common and the rate of microcephaly in ASD may not differ from the average population (Lainhart et al., 2006). In addition to ASD, macro- and microcephaly have been observed in a large number of other human syndromes (Pirozzi et al., 2018). Macrocephaly often corresponds with megalencephaly, i.e., enlargement of the brain disproportionate to the height of the patient. Indeed, the Autism Phenome Project, an ongoing longitudinal study that begun in 2006, found that 15% of boys and 4% of girls with ASD had megalencephaly (Amaral et al., 2017).

**Figure 1:**
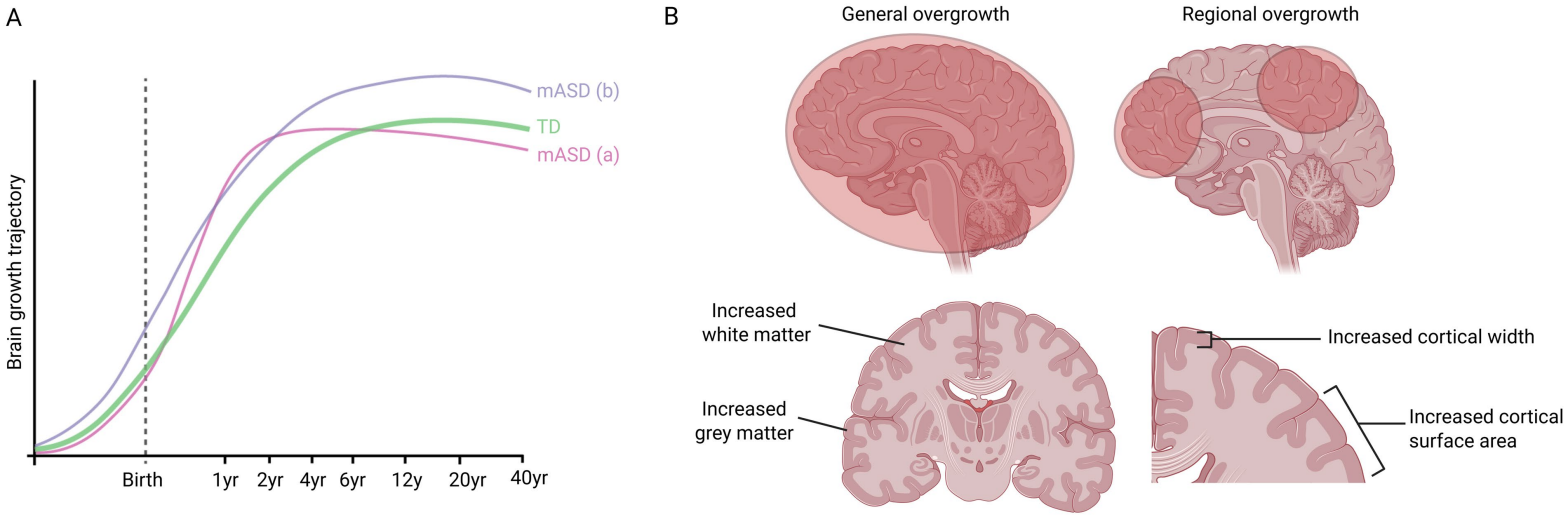
Possible brain growth trajectories of people with macrocephalic ASD (mASD) compared to typically developing (TD) people. **(A)** One possible trajectory (a) involves precocious growth during early childhood, before regression during later childhood/adolescence. Another possible trajectory (b) involves overgrowth beginning during embryogenesis and continuing throughout life. **(B)** Studies of ASD have pointed to general overgrowth of the frontal, temporal and parietal lobes, while other studies have identified regional overgrowth, e.g., of the amygdala or hippocampus. Brain enlargement can also be due to increased white matter, increased grey matter, or both. In addition, some studies have found increased surface area but not thickness of cortex, while other studies found that the cortex was thicker.

A leading theory postulates that a subset of people with ASD display precocious brain growth during early childhood followed by a subsequent regression of brain volume to a normal sized brain by adolescence (Redcay and Courchesne, 2005). This theory is based on findings of enlarged brains in young children with ASD (Courchesne et al., 2001; Courchesne et al., 2003; Hazlett et al., 2005; Sparks et al., 2002) and an absence thereof in adolescents or adults (Aylward et al., 2002). However, the transient enlargement theory is primarily based upon cross-sectional research, which can be prone to sampling bias, pointing to the need for longitudinal studies. Indeed, the Autism Phenome Project found that boys with ASD and disproportionate macrocephaly continue to have enlarged brains until at least 13 years of age (Libero et al., 2016; Lee et al., 2021). Similarly, other studies have also found increased brain volume in adolescents and adults with ASD (Hazlett et al., 2006; Hardan et al., 2001; Piven et al., 1995; Freitag et al., 2009). A meta-analysis of 44 MRI and 27 head circumference studies of idiopathic ASD patients found brain overgrowth and macrocephaly in ASD across all ages, although it was most pronounced at early ages (Sacco et al., 2015). Moreover, while some studies show that the head size of children with ASD is not altered or is slightly smaller at birth (Courchesne et al., 2001; Hazlett et al., 2005; Chiu et al., 2007), other studies find that brain overgrowth can be detected *in utero* (Bonnet-Brilhault et al., 2018). In summary, the trajectory of brain overgrowth remains unclear and requires more investigation. It is likely that the growth trajectory of macrocephalic ASD is heterogeneous, with some patients displaying macrocephaly only prenatally, only during childhood, or throughout life (Figure 1A).

In addition to the complexity of the developmental trajectory of brain overgrowth, its neuroanatomy is also multi-faceted. Some studies have pointed to general overgrowth of the frontal, temporal and parietal lobes (Redcay and Courchesne, 2005; Hazlett et al., 2005; Sparks et al., 2002), while other studies identified that only specific structures, such as the amygdala or hippocampus were affected (Schumann et al., 2004). Moreover, brain enlargement can be due to increased white matter, increased grey matter, or both (Courchesne et al., 2001; Hazlett et al., 2005; Freitag et al., 2009; Calderoni et al., 2012; Schumann et al., 2010; Palmen et al., 2005). Furthermore, while some studies found increased surface area but not thickness of cortex (Ohta et al., 2016), other studies found that the cortex was thicker. For instance, a recent large study of 1,327 MRI scans (491 with ASD) found widespread increased cortical thickness in ASD, particularly in the frontal and superior temporal cortex, precuneus, and posterior cingulate cortices (Bedford et al., 2020). Collectively, these studies show that even within the macrocephalic ASD subgroup, there is anatomical heterogeneity, which, when combined with the genetic heterogeneity (see below), suggests that there may be several different processes influencing brain size in ASD (Figure 1B).

## 3 Genetics of macrocephalic ASD—common pathways

**Figure 2:**
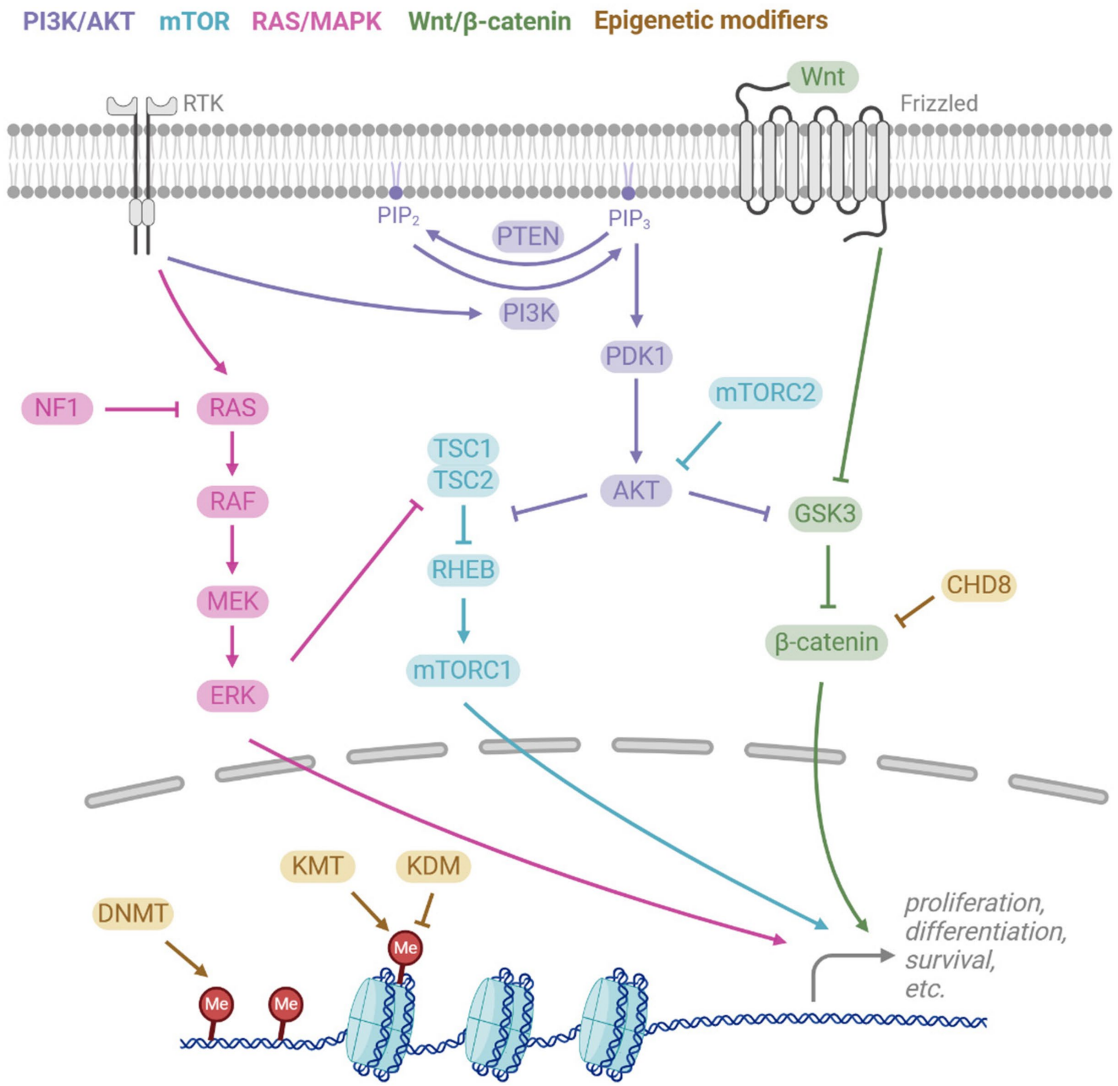
Key signalling pathways implicated in macrocephalic ASD. The PI3K/AKT pathway is activated by receptor tyrosine kinases (RTKs), activating PI3K which converts PIP_2_ to PIP_3_. PTEN antagonises this by reversing the conversion. PIP_3_ leads to downstream activation of AKT, which then regulates the mTOR and Wnt/β-catenin pathways. RTKs also activate the RAS/MAPK pathway, ultimately leading to activation of ERK which also regulates mTOR. The canonical Wnt signalling pathway involves the binding of Wnt to the receptor Frizzled, resulting in inhibition of the β-catenin destruction complex, which includes GSK3, and hence Wnt binding activates β-catenin activity. All pathways play important downstream roles in cell functions such as proliferation, differentiation, and survival. Epigenetic modifiers also regulate transcription of these pathways, acting via DNA methyltransferases (DNMT), histone lysine methyltransferases (KMT), histone lysine demethyltransferases (KDM), and chromatin remodelers (e.g., CDH8).

While ASD is genetically complex, several signaling and epigenetic pathways are frequently implicated in macrocephalic ASD, which when mutated are often highly penetrant, resulting in syndromes with macrocephaly and ASD (Tables 1, 2). These pathways are also highly pleiotropic and regulate many aspects of neural development, such as progenitor proliferation, differentiation and cell death. Here, we will introduce some of the major pathways implicated in macrocephalic ASD (Figure 2). In later sections, we will discuss how these pathways and others influence biological processes which may lead to macrocephalic ASD.

**Table 1:** PI3K-AKT–mTOR, RAS-MAPK, and Wnt-β-catenin pathway-associated genes causing macrocephaly and ASD.

Pathway	Gene	Role in pathway	Evidence in macrocephaly and ASD
PI3K-AKT–mTOR	PTEN	Core member	Klein et al. (2013)
	TSC1 and TSC2	Core member	Jeste et al. (2016)
	RHEB	Core member	Reijnders et al. (2017)
	mTOR	Core member	Yeung et al. (2017), Mirzaa et al. (2016), Baynam et al. (2015), and Smith et al. (2013)
	RAB39b	Interacts with PI3K-AKT signalling (Zhang et al., 2020)	Giannandrea et al. (2010)
	PP2A family (e.g., PPP2R5D)	Negatively regulates AKT (Loveday et al., 2015)	Houge et al. (2015) and Loveday et al. (2015)
Wnt/β-catenin	WDFY3 (i.e., ALFY)	Regulates Wnt (Kadir et al., 2016)	Le Duc et al. (2019)
	SHANK3	Directly interacts with β-catenin at the synapse (Hassani Nia et al., 2020)	Sarasua et al. (2023)
RAS-MAPK	NF1	Negatively regulates RAS signalling (Sanchez-Ortiz et al., 2014)	Greenwood et al. (2005)
	RAF (e.g. BRAF)	Core member	Allanson et al. (2011)
	MEK (MEK1, MEK2)	Core member	Allanson et al. (2011)
	RAS (e.g., HRAS)	Core member	Gripp et al. (2010)
	TAOK1 (MAP3K)	Core member	Cavalli et al. (2024)
	SHOC2	Forms a complex with PPP1CB and MRAS to activate RAF (Young et al., 2018)	Komatsuzaki et al. (2010)
	PPP1CB	Forms a complex with SHOC2 and MRAS to activate RAF (Young et al., 2018)	Gripp et al. (2016)
Histone lysine methyltransferase	KMT2C	Methylation of H3K4 (Shen et al., 2014)	Siano et al. (2022)
	KMT2E	Indirectly regulates methylation of H3K4 (Shen et al., 2014)	O’Donnell-Luria et al. (2019)
	KMT5B	Dimethylation of H4K20 (Wickramasekara and Stessman, 2019)	Sheppard et al. (2023)
	SETD2	Trimethylation of H3K36 (Pfister et al., 2014)	Luscan et al. (2014), Lumish et al. (2015), and Zhang et al. (2023)
	NSD1	Dimethylation of H3K36 (Hamagami et al., 2023)	Tatton-Brown and Rahman (2007) and Lane et al. (2017)
Histone lysine demethyltransferase	KDM6B	Demethylation of H3K27me2/3 (Jones et al., 2018)	Rots et al. (2023)
DNA methylase	DNMT3A	*De novo* methylation of 5-methylcytosine (Gao et al., 2020).	Tatton-Brown et al. (2018) and Lane et al. (2020)
Chromatin remodelers	ARID1B	Member of the BAF complex (Moffat et al., 2019)	Vals et al. (2014) and D'Gama et al. (2015)
	CHD8	Negatively regulates transcription of β-catenin target genes (Nishiyama et al., 2012)	Weissberg and Elliott (2021) and Bernier et al. (2014)
	CHD3	Component of the NURD complex (Hoffmeister et al., 2017)	Snijders Blok et al. (2018)

See also Table 2 for more associated syndromes.

**Table 2:** Macrocephalic ASD syndromes discussed in this review.

Disorder	Gene	OMIM#	Common symptoms	Sources
PTEN hamartoma tumor syndromes (including Cowden syndrome, Bannayan-Riley-Ruvalcaba syndrome, PTEN-related Proteus syndrome, and PTEN-related Proteus-like syndrome, Lhermitte-Duclos disease)	PTEN	158,350	Benign tumour-like growths (hamartomas) Intellectual disability Overgrowth Increased risk of cancer Macrocephaly ASD	Macken et al. (2019)
Smith-Kingsmore syndrome	MTOR	616,638	Macrocephaly Intellectual disability Seizures ASD	Baynam et al. (2015), Smith et al. (2013), Poole et al. (2021)
16p11.2 recurrent deletion syndrome	Deletion of ~600 kb segment on chromosome 16	611,913	Motor speech disorder Language disorder ASD Obesity Macrocephaly	Kumar et al. (2008), Steinman et al. (2016)
Sotos syndrome	NSD1	117,550	Macrocephaly Overgrowth Learning disability ASD	Tatton-Brown and Rahman (2007) and Lane et al. (2017)
Luscan-Lumish syndrome	SETD2	616,831	Overgrowth Macrocephaly Speech delay Chiari I malformation ASD	Luscan et al. (2014), Lumish et al. (2015), and Zhang et al. (2023)
Fragile X syndrome	FMR1	300,624	Intellectual disability Developmental delay ASD Macrocephaly	Kaufmann et al. (2017) and Chiu et al. (2007)
Neurofibromatosis type 1 (NF1)	NF1	162,200	Benign tumors in nerves and skin Pigmentation of skin Bone disorders ASD Macrocephaly	Garg et al. (2013) and Basto et al. (2022)
Tuberous sclerosis complex	TSC1 or TSC2	191,100 613,254	Benign tumors in skin, brain, and other organs. Seizures ASD Increased risk of macrocephaly	Vignoli et al. (2015), Fidler et al. (2000), and Levine et al. (2023)
Phelan-McDermid syndrome	SHANK3 or deletions of the 22q13 genomic region	606,232	Developmental delay Neonatal hypotonia ASD Macrocephaly or microcephaly	Sarasua et al. (2023) and Oberman et al. (2015)
Cortical dysplasia focal epilepsy (CDFE) syndrome	CNTNAP2	610,042	Intellectual disability Speech impairment Seizures ASD Macrocephaly	Strauss et al. (2006), de Jong et al. (2021) and Peñagarikano and Geschwind (2012)
Intellectual developmental disorder, autosomal dominant 51 (MRD51)	KMT5B	617,788	Developmental delay Macrocephaly Autism Congenital abnormalities	Sheppard et al. (2023)
O’Donnel-Luria-Rodan syndrome (ODLURO)	KMT2E	618,512	Developmental delay Intellectual disability ASD Macrocephaly	O’Donnell-Luria et al. (2019)
Coffin-Siris syndrome	BAF complex genes, most commonly ARID1B	135,900	Developmental delay Finger or nail hypoplasia ASD or autistic traits Macrocephaly in some cases	Vals et al. (2014), Milutinovic et al. (2023), and Van Der Sluijs et al. (2019)
Tatton-Brown-Rahman syndrome	DNMT3A	615,879	Overgrowth Intellectual disability Macrocephaly ASD	Tatton-Brown et al. (2018) and Lane et al. (2020)
Intellectual developmental disorder with autism and macrocephaly (IDDAM)	CHD8	615,032	Developmental delay ASD Macrocephaly	Bernier et al. (2014)
Houge-Janssens syndrome 1 (HJS1)	PPP2R5D	616,355	Intellectual disability Hypotonia Seizures Macrocephaly ASD	Loveday et al. (2015), Houge et al. (2015), and Shang et al. (2016)
Cardiofaciocutaneous syndrome (CFC)	BRAF MAP2K1 MAP2K2 KRAS	115,150	Cardiac abnormalities Cutaneous abnormalities Overgrowth Macrocephaly ASD	Garg et al. (2017), Allanson et al. (2011), and Adviento et al. (2014)
Costello syndrome	HRAS	218,040	Developmental delay Cardiac abnormalities Short stature Macrocephaly ASD	Adviento et al. (2014) and Lin et al. (2009)
Noonan syndrome	PTPN11 SOS1 KRAS NRAS BRAF MAP2K1 MAP2K2 CBL SHOC2 RRAS2	163,950	Short stature Characteristic facial features Cardiac defects Autistic traits Macrocephaly in some cases	Naylor et al. (2023), Myers et al. (2014), Niihori et al. (2019), and Capri et al. (2019)
Intellectual developmental disorder, X-linked 72	RAB39BC	300,271	Intellectual disability ASD Epilepsy Macrocephaly	Giannandrea et al. (2010)
Stolerman neurodevelopmental syndrome	KDM6B	618,505	Developmental delay Intellectual disability ASD Macrocephaly	Rots et al. (2023)
Developmental delay with or without intellectual impairment or behavioral abnormalities	TAOK1	619,575	Developmental delay Macrocephaly ASD or autistic traits	Cavalli et al. (2024)

### 3.1 PI3K-AKT–mTOR pathway

One of the most commonly affected signaling pathways in macrocephalic ASD is the PI3K-AKT–mTOR pathway (Table 1), which has been implicated in 47.6% of patients in a cohort with ASD and macrocephaly (Yeung et al., 2017; Magdalon et al., 2017; Switon et al., 2017; Winden et al., 2018). A key member of this pathway, PTEN, is particularly prominent in macrocephalic ASD and may account for approximately 17% of macrocephalic ASD cases (Klein et al., 2013; Butler et al., 2005; Varga et al., 2009).

The PI3K-AKT pathway is activated by receptor tyrosine kinases (RTKs), such as insulin growth factor receptor (IGF-IR) and fibroblast growth factor receptors (FGFRs) (Figure 2). Ligand binding to RTKs activates PI3K, which phosphorylates the membrane lipid Phosphatidylinositol (4,5)-bisphosphate (PIP_2_) to phosphatidylinositol (3,4,5)-trisphosphate (PIP_3_). PTEN antagonises PI3K by converting PIP_3_ back to PIP_2_. PIP_3_ activates AKT, a protein kinase that interacts with several downstream signaling pathways, such as the mTOR pathway. AKT inhibits the TSC1/TSC2 complex, which are negative regulators of RHEB that activates the mTOR complex 1 (mTORC1). Thus, AKT promotes mTORC1, leading to downstream promotion of cell-cycle and growth. Another key target of AKT is GSK3, through which the PI3K-AKT pathway regulates the Wnt/*β*-catenin pathway.

mTOR is a major signaling hub that integrates signals to regulate key processes, including cell growth and metabolism. mTOR is part of two distinct complexes, mTORC1 and mTORC2, which differ in their protein composition and function. In addition to the PI3K-AKT regulation of mTORC1 via inhibition of TSC2, ERK and RSK also act on TSC2. mTORC2 positively regulates AKT, but in contrast to mTORC1, less is known about the upstream regulation of mTORC2. However, mTORC2 is known to be activated by neurotrophins, glutamate, and NMDA (Sundberg and Sahin, 2020).

### 3.2 Wnt/*β*-catenin

Canonical Wnt/*β*-catenin signaling begins with the activation of the ligand, Wnt, by the transmembrane receptor Frizzled, as well as the coreceptors LRP5/6. This triggers inhibition of the *β*-catenin destruction complex, composed of Axin, APC, CK1, and GSK3β. Wnt binding allows β-catenin to accumulate and translocate to the nucleus where it interacts with TCF/LEF transcription factors to promote genes involved in cell proliferation (Caracci et al., 2021). CTNNB1 (encoding β-catenin) is an ASD risk gene, with loss-of-function mutations in CTNNB1 can cause microcephaly (Ligt et al., 2012) and loss of function mutations to genes that antagonize β-catenin can result in macrocephaly (Table 1).

### 3.3 RAS-MAPK pathway

The RAS-MAPK pathway is well known for its role in cancer and plays important roles also in the developing brain. Mutations, typically gain-of-function, in components of the RAS-MAPK pathway are associated with a group of clinically similar syndromes known as “RASopathies,” which include neurofibromatosis type 1 (NF1), cardiofaciocutaneous syndrome (CFC), Costello syndrome, and Noonan syndrome (Table 2) (Bustelo et al., 2018). RASopathies are often characterized by intellectual disability, short stature, ASD, and macrocephaly (Garg et al., 2017; Allanson et al., 2011).

The RAS-MAPK pathway is highly interconnected with PI3K-AKT pathway (Figure 2). Both pathways are activated by the same receptors and there is parallel signaling and crosstalk between the pathways. Receptor Tyrosine Kinases (RTKs) such as FGFRs and IGF1R activate RAS, causing a MAPK phosphorylation cascade involving RAF, MEK, and ERK. ERK in turn regulates downstream targets such as transcription factors and TSC2.

### 3.4 Epigenetics

Epigenetic genes contribute to a large proportion of high-confidence ASD risk genes identified by exome sequencing studies (Satterstrom et al., 2020; Sanders et al., 2015; De Rubeis et al., 2014). In the SFARI “high confidence” gene list, epigenetic genes constitute ~23%, despite constituting only ~3.5% of protein coding genesp; a 6.5-fold overrepresentation (see footnote 1). Interestingly, many epigenetic genes are also associated with macrocephaly (Tables 1, 2).

Histone modifications involve the addition or removal of chemical groups, such as methyl or acetyl to histone proteins, altering the chromatin’s structure and accessibility. Several histone lysine methyltransferases (KMT) and demethylases (KDM) have been identified as ASD risk genes (Table 2) (Faundes et al., 2018). Moreover, heterozygous mutations in the histone methyltransferases NSD1 and SETD2 are associated with the Sotos and Luscan-Lumish syndromes, respectively. Both syndromes are childhood overgrowth disorders characterized by macrocephaly, intellectual disability, social deficits, and often ASD (Table 2) (Tatton-Brown and Rahman, 2007; Lane et al., 2017; Luscan et al., 2014; Lumish et al., 2015).

Another form of chromatin modification is DNA methylation, which alters DNA interactions with proteins. Mutations to the DNA methylating protein DNMT3A mutations cause Tatton Brown Rahman syndrome, which is associated with macrocephaly and ASD (Yokoi et al., 2020).

ATP-dependent chromatin remodelers modify the chromatin by altering the composition or position of nucleosomes. A major chromatin remodeler is the BAF complex (i.e., mammalian SWI/SNF complex), which can move or eject nucleosomes. Several members of the BAF complex are associated with ASD (Ronan et al., 2013). Furthermore, mutations to BAF proteins, most commonly ARID1B, cause Coffin-Siris syndrome which is associated with macrocephaly and ASD-related behaviors (Vals et al., 2014). Human brain organoids heterozygous for ARID1B show increased proportion of GABAergic cells (Paulsen et al., 2022). In addition, ARID1B perturbation in human brain organoids results in abnormal ventral progenitor expansion and aberrant cell fate specification (Li et al., 2023), as well as underdevelopment of the main interhemispheric axon tract, the corpus callosum (Martins-Costa et al., 2024).

The CHD family are another major group of chromatin remodelers. CHD8 is among the top genes most strongly associated with ASD, including macrocephalic ASD (Weissberg and Elliott, 2021). CHD8 plays several roles, including binding to *β*-catenin and negatively regulating its target genes, to thereby modify neuronal development, synapse formation, and axon growth. Other members of the CHD family, such as CHD2 or CHD3, have also been implicated in neurological disorders and have been associated with ASD or macrocephaly (Lamar and Carvill, 2018; Snijders Blok et al., 2018).

## 4 What underlies brain overgrowth in macrocephalic ASD?

**Figure 3:**
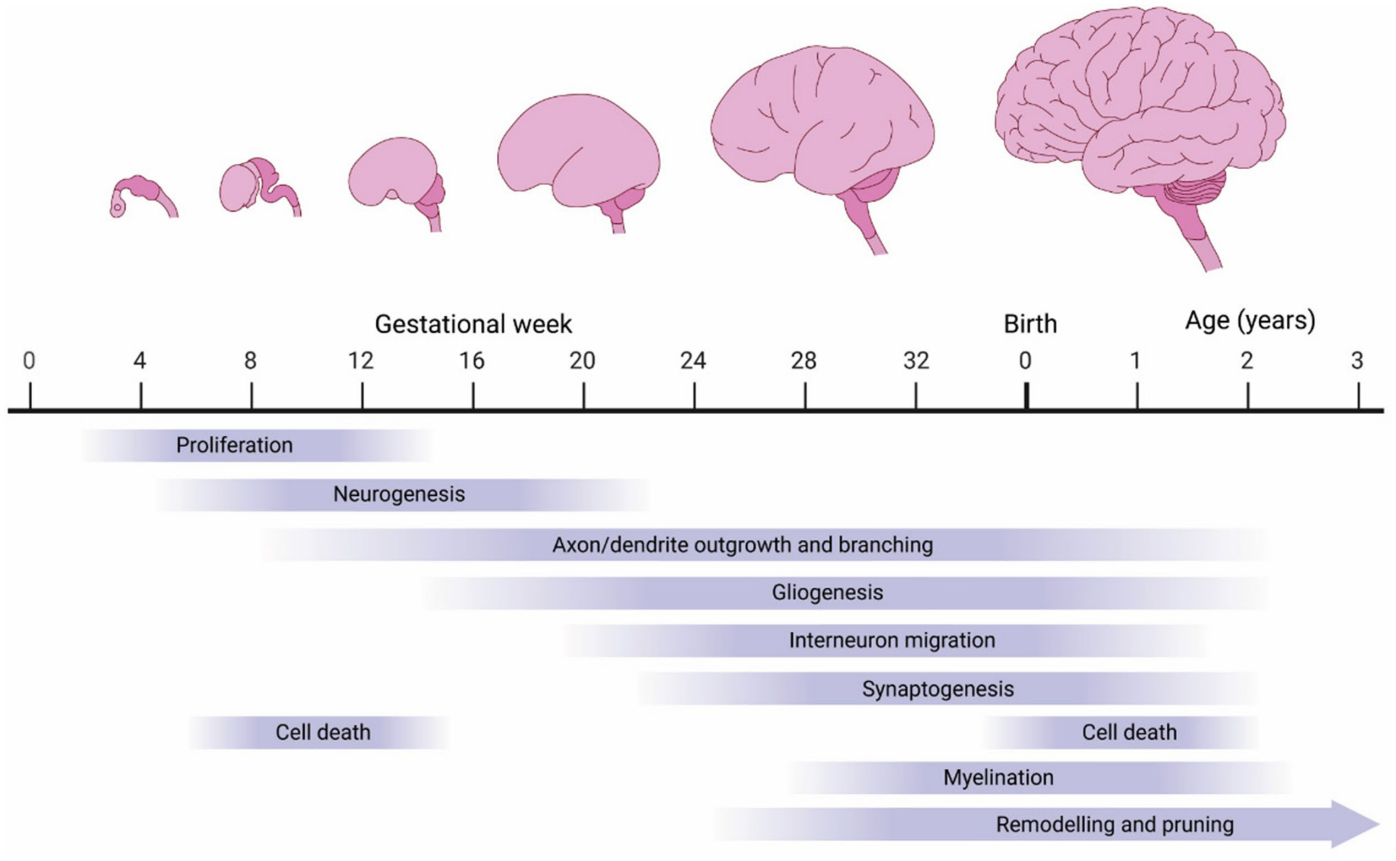
Biological processes influencing human brain growth. The human brain undergoes tremendous growth during embryogenesis, driven by proliferation (neuro- and gliogenesis). However, much of the growth occurs postnatally driven by continued axon/dendrite extensions and myelination.

Several developmental features combine to determine brain size, the most prominent of which are proliferation of neural progenitor cells, generation of neurons and glia, outgrowth of neurons and glia, cell death, myelination, synaptogenesis, and neural pruning (Figure 3). We will first review these events during normal development and then discuss how changes in these processes may contribute to macrocephalic ASD.

### 4.1 Increased proliferation

#### 4.1.1 Cortical development under normal conditions

**Figure 4:**
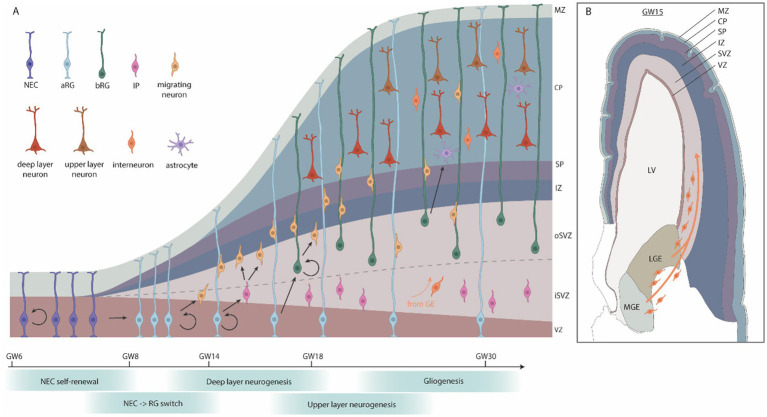
Human cortical embryonic development. **(A)** Schematic cross-section of the developing human cortex. Neuroepithelial cells (NECs) proliferate symmetrically and then give rise to apical radial glia (aRG), which generate neurons either directly, or indirectly via intermediate progenitors (IPs) or basal radial glia (bRG). IPs typically produce a few neurons while bRGs have a high capacity for self-renewal, producing many neurons. During later stages of corticogenesis, a subset of progenitor cells switches from neurogenesis to gliogenesis. **(B)** Coronal section of the brain at GW15, adapted from the BrainSpan Atlas for the Developing Human Brain (https://www.brainspan.org/static/atlas). Interneurons and oligodendrocyte progenitor cells (OPCs) migrate from the ganglionic eminences into the cortical plate. GW, gestational week; MZ, marginal zone; CP, cortical plate; SP, subplate; IZ, intermediate zone; SVZ, subventricular zone; VZ, ventricular zone; LV, lateral ventricle; LGE, lateral ganglionic eminences; MGE, medial ganglionic eminences.

In mammals, corticogenesis begins with neuroepithelial cells (NECs), an epithelial-like cell that emerges from the dorsal telencephalic vesicles (Figure 4A). At the onset of corticogenesis, at approximately gestational week 6 (GW6), NECs undergo symmetrical proliferation to expand their population, a critical step in determining the overall size of the cortex. Interestingly, evidence from cortical organoids indicate that humans display an extended symmetric NEC expansion phase when compared to apes (Benito-Kwiecinski et al., 2021), likely contributing to the increased size of the human cortex when compared to the ape cortex. Commencing at ~GW8 NECs transition from epithelial toward glial features, evident by, e.g., PAX6 expression, and become apical radial glial cells (aRGCs). Similar to NECs, aRGCs maintain apical-basal attachments, but switch to asymmetric divisions, producing one aRGC and a daughter cell of more restricted potential at each division. Daughter cells display either direct neurogenesis, where the daughter cell directly differentiates into a neuron, or indirect neurogenesis, where the daughter retains partial progenitor properties. Dividing daughter cells display a number of behaviors, but can grossly be divided into two main subtypes: intermediate progenitor cells (IPC), which lack apical or basal attachments, and basal radial glia cells (bRGs), which have basal attachments (reviewed in Thor, 2024). Both IPCs and bRGs are born at the apical surface and migrate to the basal side of the ventricular zone, where they form the subventricular zone (SVZ). Most IPCs divide once, symmetrically, to generate two neurons, while bRGs can divide multiple times, and can generate lineages of considerable size (Thor, 2024; Cárdenas and Borrell, 2020). Both IPCs and bRGs are found in the rodent, ferret and primate developing cortex, but are more prevalent in the primate cortex. Indeed, the evolutionary increase of the bRG population is generally believed to have contributed greatly to the increased size of the cortical plate, by expansion of the SVZ, and in gyrencepahlic (folded cortex) species, such as primates, the sub-division of the SVZ into an inner and outer SVZ (iSVZ and oSVZ) (Cárdenas and Borrell, 2020).

Newly generated neurons migrate into the cortical plate in an inside-out manner, with the early-born neurons forming the inner layers and late-born neurons forming the outer layers (LaMonica et al., 2012). Following neurogenesis (approximately GW20), a subset of aRGCs switch to producing glial cells, such as astrocytes and oligodendrocytes, which continue to be produced postnatally. There is evidence for ongoing adult neurogenesis in the human hippocampus (Kempermann et al., 2018). However, because macrocephalic ASD is typically diagnosed in early childhood, adult neurogenesis is unlikely to be a major driver of this phenotype.

While most cortical cells are generated inside the developing cortex (the dorsal telencephalon), some cell types arise from other regions and migrate into the cortex (Figure 4B). This includes the GABAergic, inhibitory interneurons, which are generated in the ganglionic eminences and migrate into the cortical plate during mid-to-late gestation and early infancy (Xu et al., 2011). In addition, some populations of oligodendrocyte precursor cells (OPCs) are generated in the ganglionic eminence and migrate into the cortical plate around mid-gestation (Rakic and Zecevic, 2003). In humans, there is evidence that interneurons may also arise from the cortical subventricular zone, but the extent of this is unclear (Hansen et al., 2013; Zecevic et al., 2011; Ma et al., 2013; Delgado et al., 2022).

In summary, there are four key factors that determine the number of cells in the cortex: the number of NECs generated before onset of neurogenesis, the number of neurogenic cell cycles of the aRGCs, the extent of direct versus indirect neurogenesis and the migration of cells into the cortex. Changes in any one of these four processes could lead to an increased number of cells in the cortex, and consequently, megalencephaly. Moreover, depending upon the mechanisms, increased cell numbers could contribute to the excitation-inhibition imbalance viewed as a driver ASD pathology (see section 5 below).

#### 4.1.2 Corticogenesis is altered in macrocephalic ASD

One of the leading theories for brain overgrowth in ASD is excess neurogenesis (Courchesne et al., 2019; Donovan and Basson, 2017; Packer, 2016; Kaushik and Zarbalis, 2016). This is supported by postmortem, induced pluripotent stem cell (iPSC), and animal studies. Several postmortem studies have found increased numbers of neurons or glia in ASD patients, with a 67% increase in neuronal numbers in the prefrontal cortex of boys with ASD (Courchesne et al., 2011), and increased numbers of von Economo neurons in the frontoinsular cortex of boys with ASD (Santos et al., 2011). Von Economo neurons are found in several large-brained mammals, such as humans and great apes, and may play a role in social and emotional processes. A recent postmortem study found increased neuron numbers and decreased astrocyte numbers in layer 2 of the prefrontal cortex (Falcone et al., 2021), indicating a failure of the neurogenic-gliogenic switch in aRGCs. By contrast, another postmortem study found increased glial density in layer 2 of piriform cortex (Menassa et al., 2017), suggesting that these phenotypes may be patient- and/or region-specific. Further evidence that corticogenesis is altered in ASD comes from gene expression studies in postmortem prefrontal cortex of young boys with ASD, identifying dysregulation of pathways involving cell number, cortical patterning, and differentiation (Chow et al., 2012).

Studies using iPSCs also provide compelling support for increased proliferation as a contributing factor for macrocephalic ASD. Several studies have found that NPCs derived from iPSCs of ASD patients with brain overgrowth have increased proliferation and a more rapid cell cycle *in vitro* (Wang et al., 2020; Marchetto et al., 2017; Mariani et al., 2015). This phenotype was linked to dysregulation of *β*-catenin/BRN2 transcription (Marchetto et al., 2017). Interestingly, NPCs displayed replication stress and harbored elevated DNA double-strand breaks in genes associated with ASD pathogenesis, such as cell–cell adhesion and migration (Wang et al., 2020). In addition to accelerated cell cycle, iPSC-derived cortical organoids from ASD patients displayed overproduction of GABAergic inhibitory neurons (Mariani et al., 2015). Several transcription factors involved in NPC proliferation and neural cell fate were overexpressed, including FOXG1, and inhibition of FOXG1 was able to rescue the phenotype (Mariani et al., 2015). Another study investigated gene expression in iPSC-derived cortical neurons from patients with idiopathic ASD and found that genes involved in neuronal differentiation, axon guidance, and cell migration were dysregulated (DeRosa et al., 2018).

#### 4.1.3 Signaling pathways in cortical development and macrocephaly

Many high-risk ASD genes are involved in neurogenesis, and studies on animal models have provided valuable insight into how dysregulation of key signaling pathways can alter corticogenesis, resulting in macrocephaly. For instance, loss-of-function mutations to genes that inhibit PI3K-AKT signaling, such as PTEN, lead to macrocephaly, accompanied by elevated NPC proliferation and early differentiation *in vivo* and *in vitro* (Groszer et al., 2001; Song et al., 2018; Jo et al., 2012). Mice with heterozygous PTEN mutations have brain overgrowth with excess neurons at birth and excess glia in adulthood (Chen et al., 2015). Interestingly, this phenotype was rescued by heterozygous knockout of *β*-catenin but not mTOR (Chen et al., 2015). In line with this finding, other studies show that PTEN regulates NPC proliferation/differentiation via downstream regulation of GSK3 and β-catenin (Song et al., 2018). Moreover, deletion of GSK3 in mice results in increased proliferation of NPCs at the expense of IPC generation and neuronal differentiation. This was associated with dysregulation of *β*-catenin, Sonic Hedgehog, Notch, and FGF signaling, all of which are major regulators of NPC development (Kim et al., 2009).

Studies in human brain organoids revealed that deletion of PTEN increases proliferation and results in larger and folded cerebral organoids (Li et al., 2017). Moreover, heterozygous *PTEN* mutants display abnormal developmental timing in outer radial glia progenitors (bRG) and deep-layer cortical projection neurons (Pigoni et al., 2023). PTEN mutant human cortical organoids show hypertrophy, electrical hyperactivity, enhanced proliferation, and structural overgrowth. PTEN loss hyperactivates mTORC1 and mTORC2. Interestingly, double mutants of PTEN with RPTOR or RICTOR reveals that hyperactivation of mTORC1 and mTORC2 is crucial for PTEN mutant human neural phenotypes (Dhaliwal et al., 2024).

While the above studies demonstrate that PI3K-AKT-GSK3-*β*-catenin signaling plays a critical role in cortical development, sometimes independently of mTOR, several studies in rodents have also shown that mTORC1 is critical for NPC cell cycle and neuronal differentiation (Han et al., 2008; Cloëtta et al., 2013; Magri et al., 2011), indicating that PI3K-AKT act via both mTOR and β-catenin downstream pathways. Moreover, in addition to its canonical role in the PI3K-AKT pathway, PTEN can also promote proliferation by localizing to the nucleus where it regulates chromatin stability and G0-G1 cell cycle entry (Groszer et al., 2006; Liu et al., 2017; Misra et al., 2021).

Another important gene in macrocephalic ASD – CHD8 – interacts with β-catenin, as well as other factors such as E2F, REST, and KMT2A, to regulate cell cycle, neuronal identity, and oligodendrocyte differentiation, respectively (Weissberg and Elliott, 2021; Subtil-Rodríguez et al., 2014; Katayama et al., 2016; Zhao et al., 2018). CHD8 is critical for many aspects of corticogenesis, and mice with heterozygous Chd8 mutations have brain overgrowth with increased NPC proliferation and dysregulation of genes involved in cell cycle and chromatin modification (Gompers et al., 2017; Platt et al., 2017). Moreover, in cynomolgus monkeys, CHD8 mutations lead to increased gliogenesis, resulting in macrocephaly with increased white matter (Li et al., 2023). Differences between these animal models may reflect differences in the timing of corticogenesis across different species. Studies of heterozygous CHD8 human brain organoids pointed to accelerated development of GABAergic neurons, leading to an increased proportion of these neurons (Paulsen et al., 2022).

The RAS-MAPK pathway also plays critical roles in neurodevelopment. During early cortical mouse development, loss of ERK causes an elongated cell cycle, which disrupts NPC proliferation and promotes precocious neurogenic divisions, resulting in depletion of the NPC pool and microcephaly (Pucilowska et al., 2012). Interestingly, loss of ERK2 at a later stage of cortical development impaired NPC proliferation but decreased neurogenic divisions. NPCs remained in an undifferentiated state until gliogenesis, wherein they produce a larger number of astrocytes (Samuels et al., 2008). Similarly, inhibition of mouse PTPN11 (encoding SHP2, an upstream promotor of MAPK), caused reduced neurogenesis and increased gliogenesis (Zhu et al., 2018; Ke et al., 2007), whereas activation of SHP2 in a mouse model of Noonan syndrome caused increased neurogenesis and decreases astrogenesis (Gauthier et al., 2007). Together, these studies indicate that RAS-MAPK regulates the neurogenic/gliogenic switch by promoting neurogenic divisions.

However, numerous studies also indicate that RAS-MAPK signaling is critical for promoting gliogenesis. NPCs lacking Mek1/2 fail to transition to gliogenesis, and thus astrocytes and OPCs fail to appear in mice lacking Mek1/2 (Li et al., 2012). Conversely, mice with constitutively active Mek1 have a large increase in number of astrocytes (Li et al., 2012). Moreover, deletion of PTPN11 during later embryonic stages or in oligodendrocyte lineages results in severe reduction of OPC generation and proliferation (Zhu et al., 2010; Ehrman et al., 2014), whereas gain of function mutations to PTPN11 cause higher oligodendrocyte counts (Ehrman et al., 2014). Interestingly, RAS-MAPK signaling may also regulate the generation of interneurons. NPAS1 negatively regulates MAPK signaling in progenitors of the ganglionic eminences. NPAS1 KO mice had increased ERK signaling and proliferation, resulting in generation of an excessive number of neocortical interneurons and a thicker cortex by P30 (Stanco et al., 2014). The RAS-MAPK pathway has also been shown to promote generation somatostatin positive interneurons over other interneuron lineages (Knowles et al., 2023).

In summary, the PI3K-AKT, mTOR, Wnt-*β*-catenin, and RAS-MAPK signaling pathways regulates many aspects of corticogenesis, such as proliferation of NPCs, indirect neurogenesis, the neurogenic-gliogenic switch, and the generation of neurons and glia. Dysfunction of these pathways could result in a larger cortex due to increased numbers of neurons and/or glia. While these pathways outlined above only cover a small proportion of genes associated with ASD and macrocephaly, they provide insight into how disruptions in cortical development may result in macrocephaly in ASD patients.

### 4.2 Decreased cell death

#### 4.2.1 Cell death during normal development

During development of the nervous system, cell death is extensive and essential to ensure that cell populations are the correct size. In the cerebral cortex, there are two major waves of programmed cell death – first during embryonic stages, primarily affecting progenitor cells, and again during early postnatal development, which ultimately determines the final number of neurons (reviewed by Wong and Marín, 2019). It is estimated that 30–40% of cortical glutamatergic projection neurons and interneurons are eliminated postnatally in mice (Southwell et al., 2012; Verney et al., 2000). Projection neurons and interneurons adjust their numbers in tandem (Wong et al., 2018), which is likely vital for creating the correct excitation-inhibition balance. Additionally, certain populations of cells such as Cajal–Retzius neurons and subplate neurons are almost completely eliminated (Wong and Marín, 2019). Glia also undergo developmental cell death, such as the postnatal elimination of embryonic populations of oligodendrocytes (Kessaris et al., 2006).

#### 4.2.2 Is cell death decreased in macrocephalic ASD?

While apoptosis has been associated with ASD, is it usually increased apoptosis, rather than decreased apoptosis (Wei et al., 2014). However, because several key genes associated with macrocephalic ASD have roles in regulating cell death it is possible that apoptosis is increased specifically in macrocephalic ASD. For example, PTEN is well-known as a tumor suppressor and promotes apoptosis by negatively regulating PI3K-AKT signaling (Song et al., 2012). AKT enhances cell survival by inhibiting proapoptotic proteins such as GSK3, FOXO, PCG1, and p27 (Song et al., 2012; Manning and Cantley, 2007). Similarly, ERK1/2 in the RAS-MAPK pathway has proapoptotic functions (Sugiura et al., 2021), and Wnt/*β*-catenin and mTOR pathways also have roles in apoptosis and cell survival (Trejo-Solis et al., 2021; Hung et al., 2012). Therefore, decreased apoptosis may be a contributing factor to macrocephalic ASD, especially in cases where the above signaling pathways are altered.

### 4.3 Neuronal hypertrophy

#### 4.3.1 Neuronal outgrowth under normal conditions

The brain of a newborn human is approximately 25% the size of the adult brain, and within the first 2 years of life it grows to approximately 80% the size of the adult brain (Knickmeyer et al., 2008). Because the majority of neurons have already been generated by birth, the generation of glia and the development of dendrites, axons, and synapses are the main contributors to brain size post-birth (van Dyck and Morrow, 2017). Several studies have indicated that in many cases children with ASD do not have an abnormal head size at birth, rather they develop macrocephaly by 2 years of age (Redcay and Courchesne, 2005). In these cases, excessive generation of neural processes such as dendrites, axons, and synapses may contribute to megalencephaly.

**Figure 5:**
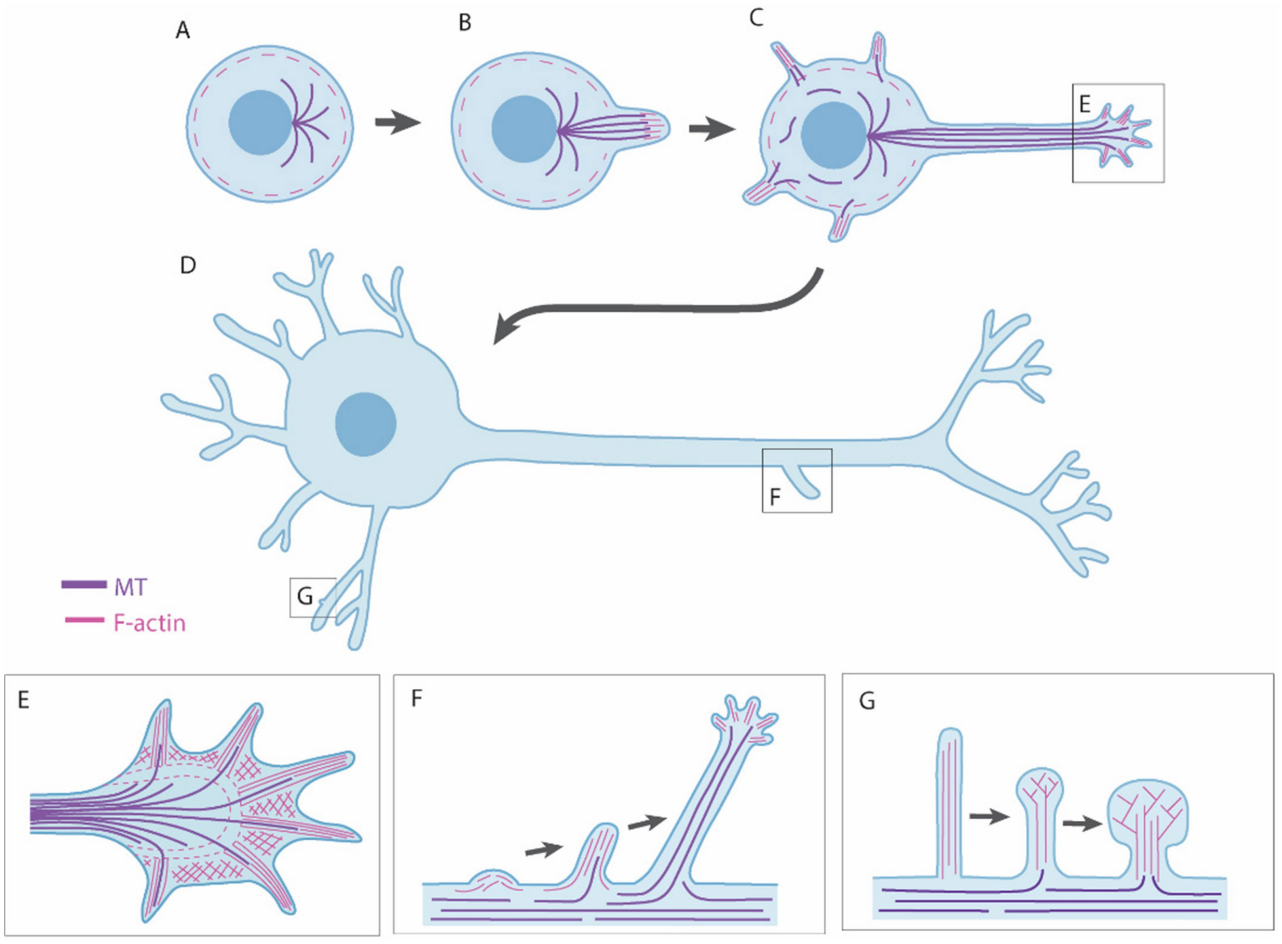
Neuronal outgrowth mechanisms. **(A,B)** Neuronal outgrowth begins with formation of neurites, caused by the formation and extension of F-actin and microtubules (MTs). **(C,D)** One neurite will form the axon and the rest dendrites. **(E)** Growth of axons and dendrites occurs at the growth cone, which has finger-like filopodia and web-like lamellipodia. **(F)** Axons and dendrites may undergo branching, which is typically caused by protrusive filopida and lamellipodia formaing along the shaft. **(G)** Dendrites form dendritic spines, in which the majority of synapses are found. Dendritic spines begin as protrusive filopodia and mature toward a mushroom-shaped sturcture as they contact and stabilize connections with other cell processes.

Axon/dendrite development commences with the outgrowth of neurites, small processes that are the precursors of axons and dendrites (Figure 5A). Neurite formation is regulated by various intrinsic and extrinsic proteins that ultimately regulate the formation, extension, and stabilization of F-actin and microtubules (MTs) (reviewed by Sainath and Gallo, 2015). Several neurites will form, but typically only one will develop a growth cone at its tip and form an axon, a process known as axon polarization (Arikkath, 2020). The axon begins extending toward its target, guided by chemorepulsive or chemoattractive signals, which either diffuse from other cells or are presented on the surface of other cells and/or extracellular matrix (reviewed by Russell and Bashaw, 2018). Most growth occurs at the growth cone, located at the distal tip of the axon. The growth cone has finger-like protrusions called filopodia, which explore the environment for guidance information. Between the filopodia are webbed-like structures called the lamellipodia (Figure 5B). Axon outgrowth results from three stages: protrusion, engorgement, and consolidation. Protrusion involves the extension of new membrane at the edges of the growth cone, driven by F-actin polymerisation at the tips of filopodia and lamellipodia. Myosin II plays an important role in this process by driving retrograde flow of F-actin, creating traction for the growth cone to advance. Engorgement involves transport of organelles and vesicles into the peripheral regions via MTs. Finally, consolidation involves the transformation of the growth cone into the axon shaft. This process is regulated by numerous actin- and MT-associated proteins (reviewed in Dent et al., 2011). Axons may also undergo branching to innervate multiple targets (reviewed in Kalil and Dent, 2014; Armijo-Weingart and Gallo, 2017). Axon branching most commonly arises from protrusive filopodia and lamellipodia which form along the shaft of the axon, independent from the growth cone (Figure 5C) (Armijo-Weingart and Gallo, 2017).

After the axon forms, other neurites begin to develop into dendrites. A distinguishing feature between axons and dendrites is that axons have one-directional MT polarity, whereas dendrites have MTs polarized in both directions to facilitate intracellular cargo transport in both directions (Yau et al., 2016). Similar to axons, dendrites have growth cones that navigate and extend through microtubule and actin polymerization. Dendrite growth is highly dynamic, undergoing constant remodeling, often branching out to cover a large area. Finally, dendritic spines are formed, which are bulbous protrusions along the dendrite where most excitatory synapses are located. Developing dendrites are covered in filopodia that extend and retract until they contact an axon, triggering them to morphologically and functionally transform into a spine (Figure 5D) (Ziv and Smith, 1996). Dendritic spines are highly dynamic and continue to form and rewire throughout development and adulthood, reflecting the plasticity of synaptic connections (Fu and Zuo, 2011).

A critical aspect of the dynamic development of axons, dendrites, and synapses is neural pruning. Axons, dendrites, and synapses are formed in abundance and then eliminated in an activity-dependent manner (Faust et al., 2021). In the human prefrontal cortex, neural pruning mostly occurs from childhood through to adolescence, but can also, to a lesser extent, extend into adulthood (Huttenlocher and Dabholkar, 1997; Petanjek et al., 2011). Pruning is regulated by numerous molecular mechanisms (reviewed by Riccomagno and Kolodkin, 2015; Schuldiner and Yaron, 2015; Faust et al., 2021). Cell death pathways such as the classical complement pathway play a major role in synaptic pruning. Moreover, axon guidance cues such as semaphorins and ephrins also regulate pruning. Microglia contribute to synaptic pruning by engulfing synaptic components in a complement-dependent manner (Schafer et al., 2012; Paolicelli et al., 2011). Astrocytes also contribute to pruning by synapse phagocytosis (Chung et al., 2013), or by secreting factors such as TGFβ that promote synaptic pruning (Bialas and Stevens, 2013).

#### 4.3.2 Neuronal hypertrophy in macrocephalic ASD

Many ASD risk genes are involved in the formation and pruning of axons, dendrites, and synapses (Satterstrom et al., 2020; Sanders et al., 2015; De Rubeis et al., 2014; Iossifov et al., 2014), suggesting that neuronal hypertrophy may contribute to macrocephalic ASD, by altering neurite outgrowth, axon/dendrite elongation, axon/dendrite branching, synapse formation, or neuronal pruning, all of which could result in increased size of the neuropil. Surprisingly, few postmortem studies have investigated axonal or dendritic arborisation in ASD patients, making it unclear to what extent this phenotype is present in macrocephalic ASD.

Increased dendrite growth or spine density has been observed in several syndromes that are often comorbid with macrocephaly and ASD. Postmortem studies indicate that patients with fragile-X syndrome have increased spine density (Irwin et al., 2000). Additionally, human iPSCs-derived neurons from patients with 16p11.2 deletion syndrome have increased soma size and dendritic length (Deshpande et al., 2017). Loss of TSC2 in human iPSC-derived neurons, modeling tuberous sclerosis syndrome, results in an increase in soma size, dendritic arborisation, and dendrite length, due to hyperactivity of PI3K-AKT pathway (Costa et al., 2016; Winden et al., 2019). Finally, it is likely that other ASD syndromes also display axonal or dendritic hypertrophy, as many genes associated with ASD syndromes, such as PTEN, mTOR, and PI3K, play major roles in axon and dendrite development (see below).

#### 4.3.3 Signaling pathways in neuronal outgrowth and macrocephaly

The PI3K-AKT pathway plays a major role in axon and dendrite formation. PTEN deletion in mouse differentiated neurons leads to macrocephaly with neuronal hypertrophy (Kwon et al., 2006), demonstrating that PI3K-AKT signaling does not only regulate brain size via neurogenesis, but also via other mechanisms. This study, along with numerous others, found that PTEN-deficient neurons have increased soma size, hypertrophic dendrites and axons (thicker with increased arborisations), and increased spine density (Kwon et al., 2006; Kwon et al., 2001; Backman et al., 2001; Gutilla et al., 2016; Gallent and Steward, 2018). This phenotype has been linked to activation of AKT/mTOR/S6K signaling and inactivation of Gsk3*β* (Kwon et al., 2006). Further studies have shown that the PI3K-AKT–mTOR pathway regulates multiple aspects of neuronal outgrowth, including soma and dendrite size (Kumar et al., 2005), dendritic branching (Kumar et al., 2005; Urbanska et al., 2012; Jaworski et al., 2005), and pruning of dendritic spines (Tang et al., 2014).

Interestingly, other studies show that the PI3K-AKT pathway regulates neuronal outgrowth via GSK3*β* rather than mTOR. PI3K-AKT-GSK3β signaling regulates MT polymerisation and stability (Kath et al., 2018; Getz et al., 2022), neurite outgrowth (Song et al., 2018), axon vs. dendrite specification (Jiang et al., 2005; Yoshimura et al., 2006), axon outgrowth (Kath et al., 2018), dendritic branching (Getz et al., 2022), and growth cone guidance (Henle et al., 2013; Chadborn et al., 2006; Meli et al., 2015). At least in some cases, this pathway functions independently of mTOR (Getz et al., 2022).

Given that GSK3*β* is an inhibitor of β-catenin signaling, PI3K/AKT likely acts by modulating Wnt/β-catenin signaling. Indeed, several studies have found roles for β-catenin in dendrite growth and spine formation (Heppt et al., 2020; Alexander et al., 2020). Interestingly, β-catenin can also regulate dendrite development by mechanisms other than its transcriptional roles, such as by interaction with the cadherin/catenin complex to regulate dendritic arborisation (Yu and Malenka, 2003), or by interacting with cadherin in dendritic spines in an activity-dependent manner to influence synaptic size and strength (Murase et al., 2002).

Interestingly, Wnts also regulate neuronal development independently of β-catenin (i.e., non-canonical Wnt signaling), including regulating axon-dendrite polarization (Zhang et al., 2007), axon guidance (Lyuksyutova et al., 2003), axon branching (Lucas and Salinas, 1997), dendritic branching (Wayman et al., 2006; Rosso et al., 2005), MT stabilization (Ciani et al., 2004), and spine formation (Ciani et al., 2011; Ramírez et al., 2016). CHD8 also plays an important role in dendrite and axon growth. Knockdown of mouse Chd8 reduces dendrite and axon growth and disrupts axon projections to the contralateral cortex (Xu et al., 2018). It is unclear whether this is through its interaction with *β*-catenin, or through its other functions.

RAS-MAPK signaling works in concert with PI3K-AKT–mTOR to regulate soma and dendrite size, axon vs. dendrite specification, dendrite complexity, and spine density (Kumar et al., 2005; Yoshimura et al., 2006; Yoshii and Constantine-Paton, 2010; Fivaz et al., 2008). Extracellular cues, such as activation of the receptor tyrosine kinase EphA8, or the cell adhesion molecule L1CAM, activates the MAPK pathway to promote neurite growth in rodents (Poplawski et al., 2012; Gu et al., 2005). Moreover, constitutively activated Ras in differentiated mouse cortical neurons or in the postnatal hippocampus leads to neuronal hypertrophy with increased soma, axon, and dendritic size, increased dendritic arborisation, increased number of synapses, and increased number of MTs per dendrite (Gärtner et al., 2004; Arendt et al., 2004; Seeger et al., 2003).

Together, these studies highlight the importance of PI3K/AKT, mTOR, Wnt/β-catenin, and RAS-MAPK signaling in neuronal outgrowth. Disruptions to these pathways could result in greater volume of the neuropil and consequently contribute to brain volume. While it is currently unclear the extent to which this occurs in idiopathic ASD, studies on these signaling pathways strongly suggests that neuronal hypertrophy may contribute to macrocephalic ASD.

### 4.4 Excess myelination

#### 4.4.1 Myelination in normal conditions

Myelin constitutes a major component of white matter, which comprises approximately 50% of the brain, raising the possibility that increases in myelination may contribute to macrocephalic ASD. Myelin is a lipid-rich multilayered membrane sheath wrapped around the axon of a neuron by oligodendrocytes and acts as an electric insulator to speed up conduction significantly. In myelinated axons, voltage-gated sodium channels are restricted to gaps between myelin sheath, known as nodes of Ranvier, resulting in a saltatory (‘leaping’) action potential (Cohen et al., 2020). Myelin is an extension of a highly compact oligodendrocyte plasma membrane, which grows by progressive wrapping of the innermost edge around the axon and coordinated lateral extension of individual layers of myelin (Snaidero et al., 2014). Myelination is regulated by cell autonomous factors in oligodendrocytes, as well as axonal factors and cues from astrocytes and microglia (Nave and Werner, 2014). Humans are born with almost no myelination in the CNS. As the oligodendrocyte population expands greatly following birth, widespread myelination occurs in first few years of childhood, and then continues to a lesser degree through adolescence into adulthood (Williamson and Lyons, 2018). Turnover of myelin is required to maintain its integrity throughout life (Meschkat et al., 2020), and adaptation of myelin sheaths contributes to nervous system plasticity (Fields, 2015).

#### 4.4.2 Can alterations in myelination cause macrocephalic ASD?

Diffusion tensor imaging (DTI) studies indicate that white matter and myelin are altered in ASD, but depending upon the study can be increased or decreased (Travers et al., 2012; Peters et al., 2012). Interestingly, the growth trajectory of white matter appears to be altered in children with ASD, with higher fractional anisotropy (FA, an indicator of white matter integrity) values at 6 months, followed by lower FA values at 24 months (Wolff et al., 2012). A limitation of these MRI studies is that they did not investigate the macrocephalic ASD subgroup specifically, making it unclear to what extent myelination is altered in macrocephalic ASD, and whether or not this contributes to brain size.

Interestingly, the PI3K-AKT, mTOR, WNT/B-catenin, and RAS-MAPK pathways are key regulators of myelination. Inactivation of PTEN in mouse oligodendrocytes results in severe myelination deficits including thickening and unraveling of the myelin sheaths (Snaidero et al., 2014; Goebbels et al., 2010; Fraser et al., 2008). Similarly, constitutive activation of AKT resulted in hyper myelination as oligodendrocytes continued myelinating throughout life (Flores et al., 2008). There was no alteration in the proliferation or death of progenitors, indicating that the PI3K-AKT directly affects myelination (Flores et al., 2008). Moreover, inhibition of Rapamycin rescued the hypermyelination phenotype, indicating that the PI3K-AKT pathway regulates myelination via mTOR signaling (Narayanan et al., 2009). Further studies showed that mTORC1 and mTORC2 regulates myelin-associated lipogenesis and protein gene regulation (Lebrun-Julien et al., 2014).

Sustained activation of ERK1/2 in oligodendrocytes also results in increased myelin thickness and expression of myelin proteins (Ishii et al., 2013; Xiao et al., 2012). Considering the similarity in phenotype with the PI3K/AKT mice, crosstalk between the pathways is likely. Indeed, while the PI3K/AKT and RAS/MAPK pathways have independent roles on the initiation and preservation of myelin, they both converge on mTORC1 signaling during active myelination (Ishii et al., 2019).

Finally, overactivation of the Wnt/*β*-catenin pathway also cause myelination deficits, however this appears to be largely due to deficits in OPC differentiation and oligodendrocyte maturation rather than a direct effect on myelin production (Fancy et al., 2009), although one study did find that Wnt/β-catenin signaling is required for myelin gene expression in zebrafish (Tawk et al., 2011).

More studies are required to determine if, and to what extent, increased myelination contributes to macrocephalic ASD. MRI studies focusing on the macrocephalic ASD cohort would be highly valuable to determine whether this subgroup have different myelination deficits compared to other ASD subgroups.

## 5 Is brain overgrowth connected to excitation-inhibition imbalance?

A leading theory for the underlying pathology of ASD is an increase in excitatory-inhibitory (E-I) ratio, resulting in abnormal brain function (Nelson and Valakh, 2015). In support of this theory, increasing neural excitation in the prefrontal cortex of mice is sufficient to cause social and cognitive disruption (Yizhar et al., 2011), although other mouse studies point to complexity in the E-I network properties involved (Antoine et al., 2019). It is possible that overgrowth of the brain in macrocephalic ASD may contribute to an imbalance of excitatory and inhibitory signaling, in several ways. First, against the backdrop of different origins of excitatory and inhibitory neurons, abnormally enhanced dorsal and/or reduced ventral telencephalon neurogenesis would likely result in excitatory-inhibitory neuron number imbalances. Such changes could stem from a dorsal expansion of the progenitor pool, enhanced indirect neurogenesis and/or prolonged neurogenesis phase, and/or the converse changes in the central area. Second, even a uniformly enhanced neurogenesis could still result in excitatory-inhibitory neuron number imbalances due to failure of later, aberrantly born, interneurons generated in the central and medial ganglionic eminences to migrate into the cortex. This may result from the gradual increase in the density of the extracellular matrix due to an abundance of axons/dendrites.

Finally, many of the genes implicated in macrocephalic ASD, such as those of the PI3K-AKT, mTOR, Wnt/*β*-catenin, and RAS-MAPK pathways, are highly pleiotropic and regulate many aspects of neural function, including synaptic function (Sánchez-Alegría et al., 2018; Maguschak and Ressler, 2012; Thomas and Huganir, 2004). Therefore, dysregulation of these genes may not only cause macrocephaly via the mechanisms discussed above, but also an imbalance of excitation/inhibition signaling. Indeed, PTEN KO mice have excitatory-inhibitory imbalance in addition to macrocephaly (Williams et al., 2015; Lugo et al., 2014; Skelton et al., 2019).

## 6 Effect of sex on megalencephaly in ASD

Males are ~4 times more likely to be diagnosed with ASD than females (Zeidan et al., 2022), and there also appears to be a higher rate of megalencephaly among males with ASD – in the Autism Phenome Project, 15% of boys but only 4% of girls displayed megalencephaly (Amaral et al., 2017). Following this, a longitudinal MRI study with 273 boys (199 with ASD) and 156 girls (95 with ASD) found that girls and boys with ASD had different growth trajectories (Lee et al., 2021). A subset of boys with ASD had disproportionate megalencephaly, and this enlargement was maintained from 2–13 years of age without volumetric regression. On the other hand, girls with ASD did not display disproportionate megalencephaly and had slower growth trajectories compared to typically developing girls (Lee et al., 2021). Sex differences in ASD prevalence does not appear to be driven by sex differences in ASD risk gene expression, but rather stem from other fundamental sex differences (Werling et al., 2016).

Low sample size may be an issue, as studies typically include fewer females than males, due to the ASD sex ratio. As such, the sample size may not be large enough to detect changes in brain size, and further studies with larger cohorts of females are required. However, if females with ASD do have lower rates of megalencephaly than males with ASD, what is the cause? Several theories have been proposed to explain the sex difference in autism. Perhaps females have lower penetrance of genetic variants associated with megalencephaly or require a greater burden of risk factors to exhibit megalencephalic ASD [see (Wigdor et al., 2022) for the female protective effect theory of ASD]. In addition, circulating sex hormones may contribute to the sexual differences in ASD prevalence. In support of this, studies in human cortical organoids and NSCs have found that androgens increase proliferation, specifically by enhancing indirect neurogenesis and resulting in increased production of excitatory neurons (Kelava et al., 2022; Quartier et al., 2018). It is tempting to speculate that if enhanced indirect neurogenesis is a common feature of male development this may “sensitise” males to macrocephaly and excitation:inhibition imbalances. Indeed, elevated testosterone levels have been linked to increased risk of ASD (Auyeung et al., 2010), although these findings are debated (Coscini et al., 2021). Altogether, it remains unclear why megalencephaly is more common in males with ASD and further studies including larger cohorts of females are needed.

## 7 Summary and future directions

As anticipated from its high prevalence, ASD is a genetically and clinically heterogeneous disorder. However, macrocephaly, often with megalencephaly, affects a substantial, 15–20%, subgroup of people with ASD, often displaying more severe symptoms (Lainhart et al., 2006; Hazlett et al., 2017). Macrocephaly may be due to excessive generation of neurons and/or glia, decreased apoptosis, excess growth of axons/dendrites, decreased neural pruning, and/or excessive myelination. While there are over 1,000 risk genes associated with ASD, the PI3K-AKT, mTOR, Wnt-*β*-catenin, and RAS-MAPK pathways, as well as epigenetics, are particularly penetrant for macrocephalic ASD. By focusing on these signaling pathways, we can gain understanding of the potential underpinnings of macrocephalic ASD. Importantly, these pathways are highly pleiotropic – they play roles at multiple developmental stages and in multiple processes. As such, it is highly likely that within an individual with macrocephalic ASD, multiple biological processes concurrently contribute to the enlargement of the brain, from proliferation through to neuronal outgrowth and synapse development.

An important step to better understand the macrocephalic subgroup of ASD is to clearly characterize its growth trajectory. Currently, the growth trajectory of brain size in macrocephalic ASD is unclear, with some studies suggesting that brain overgrowth is only present in young children with ASD, while others suggest that overgrowth continues until at least adolescence. Moreover, the actual percentage of adults with ASD that have macrocephaly is still unclear, due to a lack of studies with large sample sizes. There is a need for large high-quality longitudinal studies that track individuals from young childhood through to adult. One such is the Autism Phenome Project, which begun in 2006 and is ongoing, which will hopefully shed more light on the growth trajectory of brain size in ASD. Finally, expanded post-mortem studies, scoring neuroanatomy, cell repertoires and connectome, will also be important for understanding the underpinnings of macrocephalic ASD.

## Footnotes

^1^ https://gene.sfari.org/
